# Global analysis of T-cell groups reveals immunological features and common antigen targets of digestive tract tumors

**DOI:** 10.1007/s00432-024-05645-1

**Published:** 2024-03-15

**Authors:** Xiaoxue Li, Yuchao Zhang, Shiwei Guo, Zhenchuan Wu, Hailong Wang, Yi Huang, Yue Wang, Mengni Qiu, Jingyu Lang, Yichuan Xiao, Yufei Zhu, Gang Jin, Landian Hu, Xiangyin Kong

**Affiliations:** 1 Shanghai Institute of Nutrition and Health, CAS Key Laboratory of Tissue Microenvironment and Tumor, Chinese Academy of Sciences, Shanghai, China; 2 University of Chinese Academy of Sciences, Chinese Academy of Sciences (CAS), Beijing, China; 3 Department of Hepatobiliary Pancreatic Surgery, Changhai Hospital, Shanghai, China; 4 Anda Biology Medicine Development (Shenzhen) Co., Ltd., Shenzhen, China; 5 School of Life Science and Technology, ShanghaiTech University, Shanghai, China

**Keywords:** TCR, Tumor antigen, Digestive tract cancer, GLIPH2, Tumor immune microenvironment, Immunotherapy, Cross-reactivity

## Abstract

**Background:**

T cells are key players in the tumor immune microenvironment (TIME), as they can recognize and eliminate cancer cells that express neoantigens derived from somatic mutations. However, the diversity and specificity of T-cell receptors (TCRs) that recognize neoantigens are largely unknown, due to the high variability of TCR sequences among individuals.

**Methods:**

To address this challenge, we applied GLIPH2, a novel algorithm that groups TCRs based on their predicted antigen specificity and HLA restriction, to cluster the TCR repertoire of 1,702 patients with digestive tract cancer. The patients were divided into five groups based on whether they carried tumor-infiltrating or clonal-expanded TCRs and calculated their TCR diversity. The prognosis, tumor subtype, gene mutation, gene expression, and immune microenvironment of these groups were compared. Viral specificity inference and immunotherapy relevance analysis performed for the TCR groups.

**Results:**

This approach reduced the complexity of TCR sequences to 249 clonally expanded and 150 tumor-infiltrating TCR groups, which revealed distinct patterns of TRBV usage, HLA association, and TCR diversity. In gastric adenocarcinoma (STAD), patients with tumor-infiltrating TCRs (Patients-TI) had significantly worse prognosis than other patients (Patients-nonTI). Patients-TI had richer CD8+ T cells in the immune microenvironment, and their gene expression features were positively correlated with immunotherapy response. We also found that tumor-infiltrating TCR groups were associated with four distinct tumor subtypes, 26 common gene mutations, and 39 gene expression signatures. We discovered that tumor-infiltrating TCRs had cross-reactivity with viral antigens, indicating a possible link between viral infections and tumor immunity.

**Conclusion:**

By applying GLIPH2 to TCR sequences from digestive tract tumors, we uncovered novel insights into the tumor immune landscape and identified potential candidates for shared TCRs and neoantigens.

**Supplementary Information:**

The online version contains supplementary material available at 10.1007/s00432-024-05645-1.

## Introduction

Digestive tract tumors, including esophageal, gastric, pancreatic, liver, biliary tract, and colorectal cancers, are a group of malignant tumors with high incidence (20%) and mortality rates (35%) (Nagtegaal et al. 2020; Gonzalez et al. 2021). The development and progression of these tumors are profoundly influenced by the tumor immune microenvironment (TIME) (Rao et al. 2019). Patients’ prognosis and response to adjuvant chemotherapy are also associated with the signatures of the TIME (Jiang et al. 2018a, b; Ascierto et al. 2020; Hatogai et al. 2020; Yang et al. 2023).

T cells are essential components of the TIME because they are able to recognize and eliminate cancer cells that contain neoantigens resulting from somatic mutations. T-cell receptors (TCRs) consist of α and β chains, which are produced by the somatic recombination of different V, D, and J gene segments, forming a highly diversified TCR repertoire. Almost every naive T cell has a unique TCR sequence (Jenkins et al. 2010). TCR structure, composition (The genes that make up TCR, such as TRBV1, TRBJ1, etc.), TCR diversity, V(D)J gene rearrangement, and characteristics such as physicochemical properties of amino acids in the sequence and TCR clone expansion are key factors that affect T-cell function and fate (Lagattuta et al. 2022; Dash et al. 2017). The diversity and clonality of the TCR repertoire have been shown to be associated with the prognosis and immune therapy response of digestive tract and other solid tumors (Han et al. 2020; Hopkins et al. 2018; Sanz-Pamplona et al. 2020). The diversity and specificity of TCRs that recognize the tumor mutations in pan-digestive tract tumors are largely unknown.

The TCR consists α and β chains. Each chain is divided into a constant region (C region) and a variable region (V region), and each chain contains three highly variable regions: CDR1 (Complementarity Determining Regions), CDR2 and CDR3). Both CDR1 and CDR2 are encoded by the V-gene, and CDR3 is encoded by part of the V gene, the D gene, and part of the J gene. CDR3 is the region of the TCR that directly contacts the antigenic peptide, and plays a decisive role in the binding of the TCR to the pMHC complex; at the same time, CDR3 is also the region with the highest degree of variability, and virtually determines the diversity of the TCR.

There are a large number of CDR3 sequences and the same peptide major histocompatibility complex (pMHC) can be recognized by hundreds or thousands of different TCR sequences (Wooldridge et al. 2010). Although high-throughput sequencing technologies enable the identification of millions of TCRs, it still needs a systematic way to organize groups of TCR sequences according to their likely antigen specificities, which is beneficial for the finding of links of TCRs and their antigens. To transform this large sequence diversity into small numbers of groups with specificity to antigens, we need to use advanced algorithms and tools to cluster CDR3 sequences and reveal their shared specificity and functional correlations. GLIPH2 (Huang et al. 2020) is an algorithm based on the grouping of lymphocyte interactions by paratope hotspots that can rapidly parse millions of TCR sequences into clusters with identical or similar antigen specificity. GLIPH2 utilizes the presence of amino acid sequence motifs or strong homology within the CDR3 of TCRβ chain to infer whether two TCRs are likely to recognize the same pMHC. The contact between TCR and pMHC mostly were in the CDR3s (Tsuchiya et al. 2018). During the contact, there are multiple cases in which no CDR3α is made but at least one CDR3β sequence is required in TCR-pMHC complexes, suggesting that the latter is required, although typically both are involved (Glanville et al. 2017). This indicates that sequence analysis focused entirely on high probability contact sites in CDR3 may provide a means of clustering TCRs by shared specificity. In a previous study on TCRs in lung cancer, GLIPH2 was used to divide TCRs and identify relevant responsive TCRs (Chiou et al. 2021).

In this study, we aimed to reveal the TCR repertoire response in pan-digestive tract tumors. The transcriptome sequencing data of six digestive tract tumors from The Cancer Genome Atlas (TCGA) database was used to extract CDR3β sequences using TRUST4 (Song et al. 2021), and clustered using GLIPH2. We explored the similarities and differences between the TCR in different types of digestive tract tumors and further inferred the possible antigen types and sources that they might recognize. This work provides a theoretical basis for TCR-based immunotherapy against digestive tract cancers.

## Materials and methods

### Data and samples resource

We selected six digestive tract cancer projects from the GDC website (GDC (cancer.gov)), chose the BAM file format, and downloaded the manifest. We use the official tool gdc-client to download the corresponding bam file: Access Data | NCI Genomic Data Commons (cancer.gov). Genome versions use GRCh38. Total RNA bam files containing 1,862 samples including six types of digestive tract tumors (ESCA, *N* = 171; STAD, *N* = 402; LIHC, *N* = 424; PAAD, *N* = 182; COAD, *N* = 510; READ, *N* = 173). There were 1706 tumor tissue samples, 148 adjacent tissue (NAT) samples, 3 metastatic samples and 5 recurrent samples. The BAM files were used for the analysis described below. Cancer RNA-seq gene expression data were downloaded from UCSC Xena (xenabrowser.net). The TCR-binding peptide dataset was obtained from VDJdb (cdr3.net). VDJdb is a comprehensive antigen-specific TCR sequence database obtained by manually collecting published studies (Shugay et al. 2018). We downloaded the human CDR3 sequence from the VDJdb website. The data include information on CDR3 sequences, bound MHC epitopes, and antigen epitopes. TCGA clinical data (e.g., age, sex, survival time) were obtained from cBioPortal for Cancer Genomics via browser. The somatic mutation data of all six cancer were download at website GDC.

### The subtype of pancreatic cancer determination

The cancer subtypes of the clinical data for ESCA, STAD, COAD and READ can be obtained from the GDC website. However, pancreatic cancer subtypes were not included. Based on the gene expression markers and molecular subtypes of pancreatic cancer proposed by Collisson et al. (2011), we performed hierarchical clustering of the gene expression FPKM values of patients with PAAD and classified them into three subtypes: Exocrine-like, Classical and Quasi-mesenchymal. The R package pheatmap was used, with cutree_cols = 3, corresponding to the three molecular subtypes (Figure S9).

### HLA-type determination

The hla-genotyper (hla-genotyper · PyPI) designed by John J. Farrell is a tool used to predict HLA genotypes from RNA Seq and DNA Seq data. hla-genotyper was used to identify 1,702 HLA molecular subtypes in patients with gastrointestinal cancers, including HLA I: A, B, C and HLA II: DQA1, DQB1, DRB1. All parameters use the software defaults. Here, although most of the typing results were unique, there were a small number of patients who had multiple samples (primary tumor, adjacent to cancer, etc.). Multiple samples correspond to multiple BAM files. The prediction of HLA is inconsistent in some of these patients. We screened these patients for HLA typing based on the match quality in the output of software (qual: pass > ambiguous > low coverage).

### Calculating immune score and immune cell types

The gene expression RNA-seq data were downloaded from the UCSC database. Totally 1,854 samples were used for the analysis. The ESTIMATE algorithm was used to calculate the stromal/immune/Estimate scores and tumor purity using the “estimate” package in R (Yoshihara et al. 2013). CIBERSORTx is an analytical tool from the Alizadeh Lab and Newman Lab that imputes gene expression profiles and provides an estimation of the abundances of member cell types in a mixed cell population (Newman et al. 2015) (online website availability: CIBERSORTx (stanford.edu)). All parameters use the software defaults.

### Establishment of T-cell groups

TRUST4 is a computational tool used to extract TCR sequences from RNA sequencing data profiled from solid tissues. Information regarding the samples is provided in the supplementary Tables S1 and S2. We use TRUST4-1.0.12 for analysis. We extract the report file in the TRUST4 output for subsequent analysis, which contains the CDR3 sequences of BCR and TCR. Next, we extracted the TCR sequences from these reports, removed out_ of_frame sequence, and left only the CDR3β sequence of amino acids for subsequent analysis.

The GLIPH2 algorithm was implemented to establish T-cell groups using 107,308 CDR3β sequences. All parameters use the software defaults. The output of the CDR3β groups with shared sequence motifs was accompanied by multiple statistical measurements to facilitate the calling of high-confidence specificity groups, including biases in Vβ gene usage, CDR3β length distribution (relevant only for local motifs), cluster size, HLA allele usage, and clonal expansion. V-gene enrichment and CDR3 length distribution enrichment analyses were performed by calculating the Simpson diversity index for V genes/CDR3-lengths within clonal members of GLIPH specificity groups, and calculating the probability that a random selection of TCR sequences of the same size would generate a Simpson score equal to or greater than the observed score. Clonal expansion refers to the cloning expanded of a group. The calculation method is based on the probability of having a higher count than a randomly sized dataset. HLA enrichment was calculated for each HLA allele found in at least two members of the GLIPH convergence group (Glanville et al. 2017).

To establish high-confidence specificity groups, we prioritized TCR specificity groups with at least three distinct CDR3β members from a minimum of three different patients with significant biases in Vβ gene usage as previously described (Chiou et al. 2021). The clonal-expanded groups were filtered by the requirement of an expansion score < 0.05. The tumor-infiltrating groups were identified according to the group that only had tumor samples.

### Identification of the gene expression makers of each tumor-infiltrating cluster

Using DESeq2 (Love et al. 2014), we analyzed the differential expression using 375 tumor samples and 148 NAT samples. 375 samples were from tumor-infiltrating groups and 148 samples were all NAT samples. We first conducted differential expression analysis using these samples to identify highly expressed genes in the tumor-infiltrating groups. The counts data were normalized using the built-in function. The genes that were highly expressed in tumor samples were selected with adjusted *p* < 0.05 and |logFC|> 1. We used DESeq2 to correct for the potential bias introduced by the combination of tumor and normal samples. These genes count data was used to search for marker genes in each group using the FindMarkers function in the Seurat package of R. We also performed an alternative analysis to identify markers in each group without using differential genes between tumor and normal samples. And take the intersection of two results.

### Annotation of TCRs of clonal-expanded groups

Publicly available databases of CDR3 and antigen sequences was used to annotate the groups of patients with digestive tract cancer. The tetramer database contains CDR3β sequences that are mainly specific for viral epitopes and have been experimentally verified to bind to them in their respective HLA contexts. All tetramer data were downloaded from VDJdb. Whenever any sequence in the specificity group was annotated to publicly available antigen sequence data, the group was considered annotated, and specificity was assigned based on the relevant CDR3 sequence as well as HLA restriction. Cytoscape software was used to visualize the interactions between the annotation results. By annotating some of the clonally expanded groups with CDR3β sequences that were targeted toward known epitopes and were restricted by specific HLA types, we inferred the shared antigen specificity of the other TCRs in the same group.

### Evaluation of the impact of genes on immunotherapy

Patient data from three clinical trials: (1) IMvigor210 (National Clinical Trials identifiers NCT02951767 and NCT02108652) (Mariathasan et al. 2018), a single-arm trial studying locally advanced or metastatic urothelial carcinoma; (2) POPLAR (NCT01903993) (Fehrenbacher et al. 2016), a dual-arm trial of patients with non-small-cell lung carcinoma; and (3) IMotion150 (NCT01984242) (McDermott et al. 2018), a triple-arm trial of patients with renal cell carcinoma. We used the clinical data analysis method (Wu et al. 2020) mentioned by Wu TD et al. to establish a risk regression model for the top 25 genes highly expressed in the tumor-infiltrating group to evaluate the impact of these genes on immunotherapy. By dividing gene expression by values above or below the median, and then fitting the Cox proportional risk model to the censored PFS survival time using binary variables, the PFS risk ratio for each gene is calculated. Calculate the expression score for each patient in each trial using the common genes generated from each feature, as the weighted average z-score, and use the average t-statistic from the logistic regression model to weight each gene. Both data and methods can be obtained from the original article.

### Statistical analysis

Statistical analyses were performed using R Software (R version 4.2.2). All reported P values are two sided, and the significance level was set at 0.05 for all analyses unless otherwise noted. For diversity estimations, the GINI Simpson index was calculated using the repDiversity function of the immunarch R package (an R package for statistical computing with advanced analysis of T-cell receptor (TCR) and B-cell receptor (BCR) repertoires). Comparisons between the two groups were performed using the Kruskal‒Wallis test. Excluding the effect of extraneous factors on prognosis (control variables), we used the Cox proportional-hazards model for analysis. Kaplan‒Meier survival analysis between the two groups was performed using the R package survival. Differential expression analysis was performed on the sample count matrix of the samples. Enrichment analysis of mutations and subtypes was completed using the enricher R package with adjusted *p* < 0.05.

## Results

### Identifying tumor-infiltrating TCR groups in pan-digestive tract cancer

**Fig. 1:**
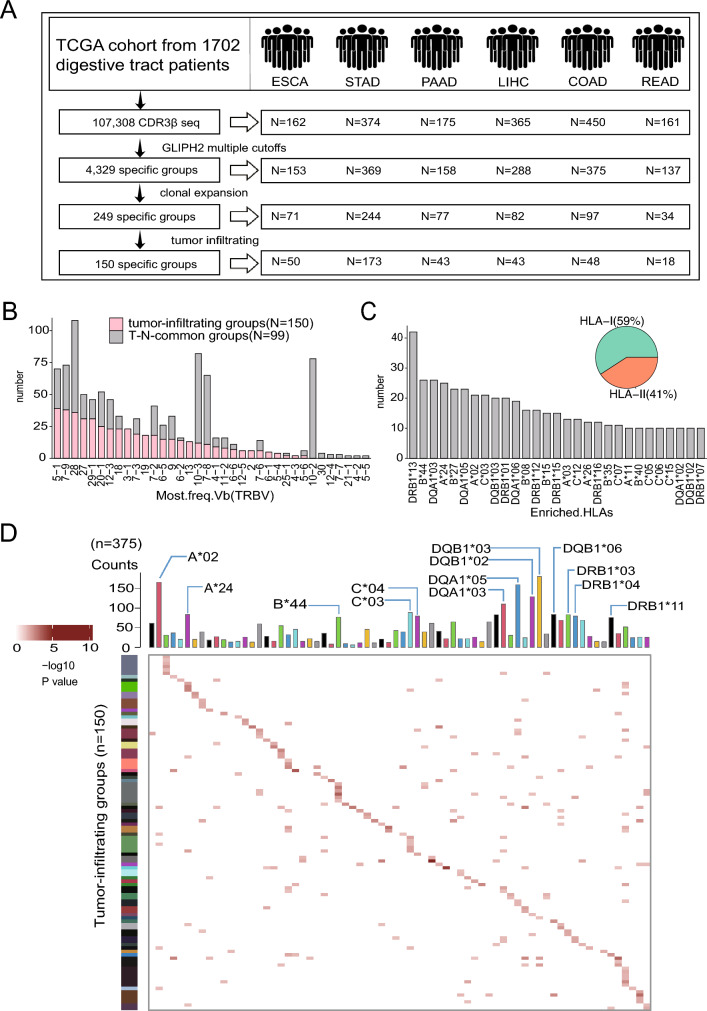
Identification of tumor-infiltrating CDR3β sequences from digestive tract cancer patients. **A** Schematic diagram of the pipeline for identifying tumor-infiltrating groups in six digestive tract cancers. **B** Distribution of the most frequent TRBV gene in each clone expansion group. The y axis shows the number of groups. The x axis shows the TRBV genes shared by those groups. **C** The number of HLAs enriched in the tumor-infiltrating group. The enriched HLA in each group is not unique. We counted the enriched HLA in all groups. **D** Heatmap of the enriched HLAs in each tumor-infiltrating group. The bar on the top shows all the HLAs in the tumor-infiltrating groups. The groups which was cluster together based on the enrichment p value is the minimum value in the group

RNA-seq data from 1,706 primary tumor samples and 148 normal samples adjacent to tumor (NAT) from surgically resected patients with pathologically confirmed esophageal carcinoma (ESCA, *N* = 162), stomach adenocarcinoma (STAD, *N* = 375), pancreatic adenocarcinoma (PAAD, *N* = 177), liver hepatocellular carcinoma (LIHC, *N* = 371), colon adenocarcinoma (COAD, *N* = 454), and rectal adenocarcinoma (READ, *N* = 163) were downloaded from the TCGA database (Table S1 and S2). We identified a total of 107,308 CDR3β sequences of TCRs from 1702 patients with digestive cancer using the TRUST4. GLIPH2 was used to cluster the TCRs by identifying CDR3β sequences with shared peptide-MHC specificity, based on local motifs and/or global homology. A total of 4329 shared groups were defined with specific filtering criteria (Vβ gene enrichment *p* < 0.05, ≥ 3 distinct CDR3β sequences and ≥ 3 distinct patients) (Fig. 1A and S1A). To focus on disease-relevant TCRs, we further identified 249 groups with evidence of clonal expansion comprising 1466 CDR3β sequences from 605 patients (clonal-expanded groups). Of these, 150 groups were specifically detected in tumors compared to NAT, comprising 699 CDR3β sequences from 375 patients (tumor-infiltrating groups) (Fig. 1A, Table S3). In these tumor-infiltrating groups, the most common V and J genes in the CDR3β sequences were TRBV5-1, TRBV7-9, TRBV28 and TRBJ2-7, TRBJ2-1. The most common V and J genes in the remaining clonal-expanded groups (T-N-common groups) were TRBV10-2, TRBV10-3, TRBV7-8, TRBV28, and TRBJ1-4 (Figs. 1B, S1B). The number of class I HLAs was higher than that of class II HLAs in tumor-infiltrating groups (142 vs 98) (Figure S1C). The most enriched HLA types in each tumor-infiltrating group were DRB1*13, B*44, and DQA1*03, the frequencies of which were overweighted in patients with tumor-infiltrating TCRs than all patients (Figs. 1C, E, and S1D). Interestingly, different tumor-infiltrating TCR groups were enriched in the same HLA type, and a given group could also be enriched in multiple HLA types (Fig. 1D, Table S4). This result indicates the low affinity and increased degeneracy of the interaction between tumor-infiltrating TCRs and HLA types.

### High diversity and poor survival in tumor-infiltrating TCR groups and clonal-expanded TCR groups

**Fig. 2:**
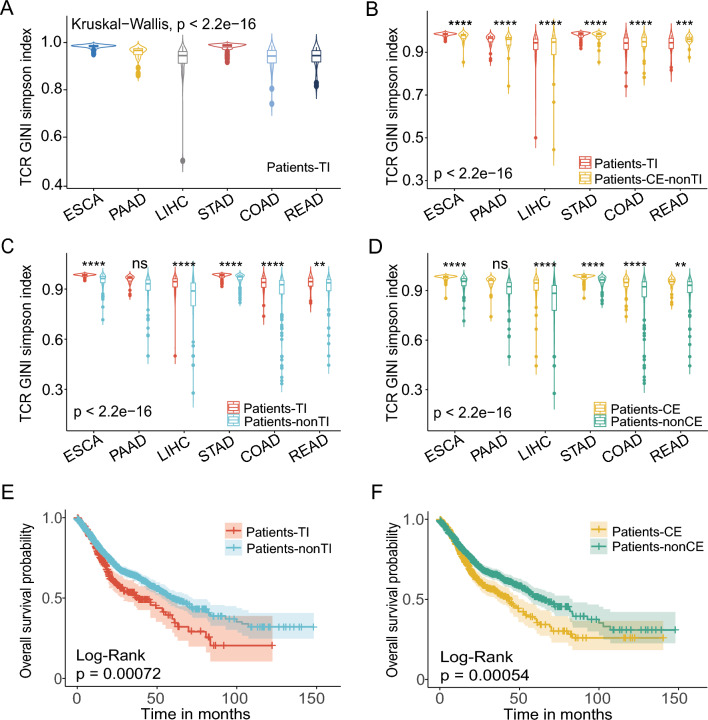
Diversity and prognosis of different cohorts.** A** The GINI Simpson index of six cancer in Patients-TI group. (Wilcox-test: *: *p* ≤ 0.05; **: *p* ≤ 0.01; ***: *p* ≤ 0.001; ****: *p* ≤ 0.0001); ****: ESCA and PAAD/LIHC/COAD/READ; STAD and PAAD/LIHC/COAD/READ; ***: PAAD and LIHC; **: PAAD and COAD; *: PAAD and READ. **B**–**D** The GINI Simpson index of different cohorts (Wilcox-test: ns: *p* > 0.05; *: *p* ≤ 0.05; **: *p* ≤ 0.01; ***: *p* ≤ 0.001; ****: *p* ≤ 0.0001). **E**–**F** Kaplan–Meier survival curves of different cohorts. Log-rank test was used to calculate p value

The diversity of TCRs in each sample was measured using the GINI Simpson index. The diversity in the tumor-infiltrating TCR groups differed significantly across the six types of cancers (Kruskal-test *p* < 2.2e−16) (Fig. 2A). Esophageal cancer and gastric cancer showed the highest average diversity (Fig. 2A). We divided the patients into five groups based on whether they carried at least one of the tumor-infiltrating TCRs or clonal-expanded TCRs: patients with tumor-infiltrating TCR groups (Patients-TI) and patients with non-tumor-infiltrating TCRs (Patients-nonTI); patients with clonally expanded TCR groups (Patients-CE), patients with nonexpanded TCRs (Patients-nonCE), and patients with expanded but non-tumor-infiltrating TCRs (Patients-CE-nonTI). There are a total of 1702 patients. The number of Patients-TI are 375. The remaining 1327 patients are Patients-nonTI. The number of Patients-CE are 605, The remaining 1097 patients are Patients-nonCE. And those patients who only appear in the clone-expanded group but are not in the tumor-infiltrating group are Patients-CE-nonTI, with a total of 230 patients. If a patient's TCR appears in both the clone-expanded group and the tumor-infiltrating group, it is Patients-TI, which is a progressive relationship.

Not all TCR clones are equally relevant for tumor recognition and elimination. Therefore, we classified patients based on whether their dominant TCR clones were relevant for tumor or not, and compare the TCR diversity, which is reported to be associated with prognosis. The results showed that the diversity of TCRs expanded only in tumor tissue was higher than that expanded in both normal and tumor tissues (ESCA, PAAD, STAD) (Fig. 2B). COAD, READ, LIHC were the opposite. Both Patients-TI and Patients-CE patients had higher TCR diversity than the remaining patients (Patients-nonTI and Patients-nonCE) (p < 2.2e−16, Fig. 2C–D). Although there was no significant difference in the diversity between Patients-TI and Patients-nonTI in patients with pancreatic cancer, the diversity of Patients-TI was higher than that of Patients-nonTI (Fig. 2C–D). The Kaplan‒Meier method was used to analyze patient prognosis. Both Patients-TI and Patients-CE had worse prognoses than the other patients (Patients-nonTI and Patients-nonCE) (*p* < 0.05) (Fig. 2E–F).

### Significantly worse prognosis of Patients-TI in tumor stage I of STAD

**Fig. 3:**
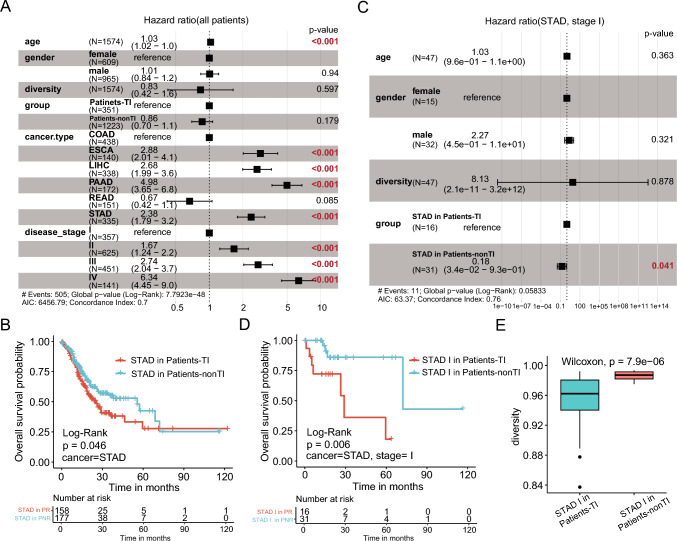
Cox proportional-hazards model and survival analysis of different cohorts. **A** and **C** Forest plot of hazard ratio of Cox proportional-hazards model adjusted for age, gender, diversity, group, cancer type and disease stage. Figure C does not include cancer type and disease stage. Log-rank *p* < 0.05 and HR < 1 indicate protective factors while HR > 1 indicate risk factors. **B** and **D** Kaplan–Meier survival curve of STAD patients. **E** Diversity comparison between two groups (Patients-TI and Patients-nonTI)

Using the Cox proportional risk model, cancer type and tumor pathology stage were found to be the most influential factors for the overall survival of patients (Fig. 3A). We performed survival analyses within each cancer type using the same Cox proportional-hazards model (Figure S2 and S3). Among them, only patients with STAD showed significant differences in prognosis between the Patients-TI and Patients-nonTI groups (Fig. 3B). This might explain the difference in prognosis between the two groups in the whole patient sample, as the sample was enriched in STAD (Fig. 2E and S4). Using the Cox proportional-hazards model that included the STAD pathology stages and the other factors (ages, sex, diversity, and group), we found that carrying tumor- TCRs was the only factor affecting prognosis in stage I but not in stages II to IV STAD patients (Fig. 3C). In stage I STAD, the patients in the tumor-infiltrating groups (STAD I in Patients-TI) had unfavorable survival (Fig. 3D), and higher diversity than the other patients (STAD I in Patients-nonTI) (Fig. 3E).

### Identifying signatures in each group of tumor-infiltrating TCR groups

**Fig. 4:**
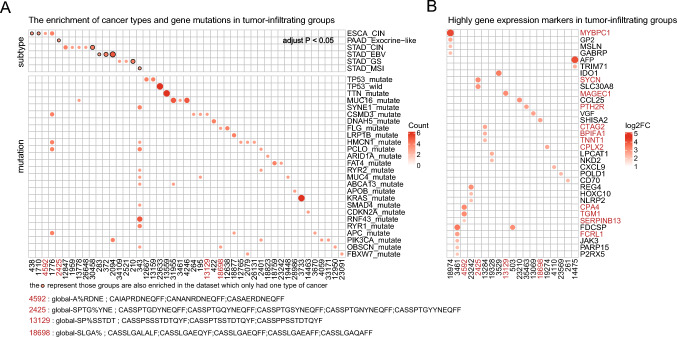
Signature of six cancer types associated with the shared groups. **A** A heatmap of gene mutations and subtype enrichment, where the columns correspond to the clustering results of GLIPH2 and the rows show the tumor subtypes (upper) and the mutated genes (down). Each dot in the heatmap indicates a significant enrichment of a subtype or gene in a cluster. The size of the dots reflects the number of enriched samples in that group. The dots with black edge represent subtypes that were also significantly enriched in individual cancer datasets. **B** A heatmap of gene markers in the tumor-infiltrating groups. markers that found used both methods were highlighted in red. Each TCR group is assigned a unique code (e.g., 4592) and consists of TCR sequences that share a common motif (e.g., A%RDNE) within their CDR3 region. The motif represents the amino acid sequence or pattern that is enriched in the TCR group relative to a reference set of naive TCRs. The percentage sign (%) indicates a position where any amino acid is allowed. The clustering method (global or local) indicates whether the TCR group is based on global similarity or local motif enrichment. Global similarity means that the TCR sequences in the group have the same length and differ at the same position, while local motif enrichment means that the TCR sequences in the group have a motif that is restricted within 3 amino acids in the CDR3 region

A multi-dimensional enrichment analysis of the patients was performed in tumor-infiltrating groups. In total, 47 groups of enrichment results for tumor subtypes and gene mutations were identified, of which 4 groups were enriched in both tumor subtypes and gene mutations (C1776: global G% DTGE; C343: global -% YAYNE; C2094: global S% VVYE; C13778: global S% GTGEG). Four of the six enriched subtypes were STAD; COAD, READ had no enriched subtype (Fig. 4A). Regarding the mutation patterns, mutated and wild-type TP53 were enriched in different groups. Moreover, tumor-related gene mutations such as TTN, MUC16, and KRAS with a higher number in groups were also enriched in distinct groups (Fig. 4A). Two groups that had specific gene expression as well as specific tumor subtypes (C4592: global A% RDNE; C2425: global SPTG% YNE) and two groups that had not only specific gene expression but also specific gene mutations (C13129: global SP% SSTDT; C18698: global SLGA%) identified (Fig. 4B). The neoantigen gene MAGE1 was highly expressed in C13129 group which also enriched the CSMD3 gene mutation. Some immune-related genes such as CCL25, CXCL9, CD70, FCRL1, and JAK3 were highly expressed in groups C23210, C4110, C261, and C3461 (Fig. 4B).

### Association of gene signatures for tumor-infiltrating TCR groups with immune-related pathways and favorable progression-free survival (PFS) in the immunotherapy arms

**Fig. 5:**
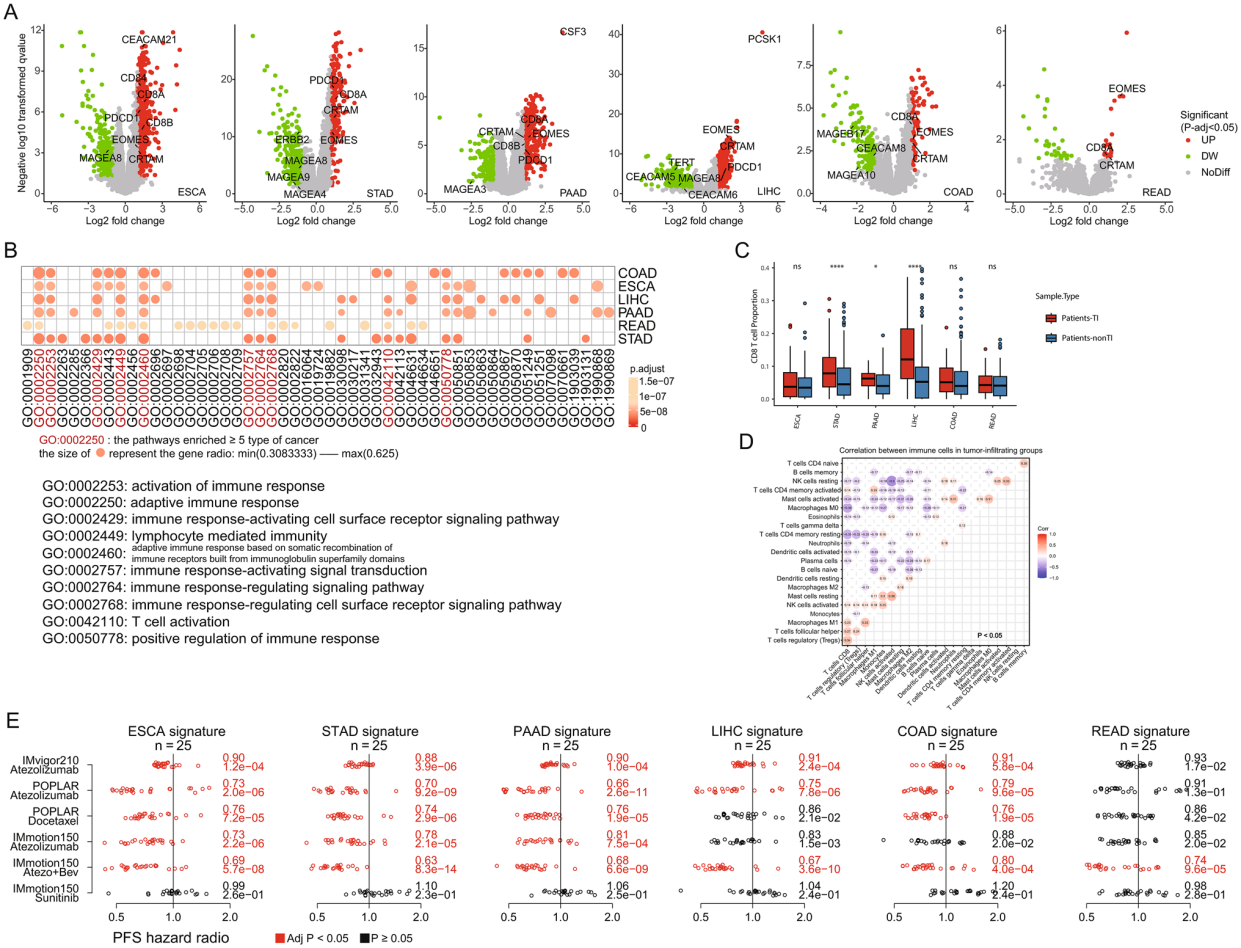
The DEG of Patients-TI and Patients-nonTI groups. **A** Differentially expressed genes (DEGs) between the Patients-TI and Patients-nonTI (|log2FC|> 1 and *p* < 0.05). **B** GO enrichment of the DEGs in all six cancers. The size of the dots in the heatmap represents the gene ratio; The larger the dot, the larger is the gene ratio. The top 20 enriched pathways are shown for each cancer type. **C** Comparison of CD8 T cell percentage between Patients-TI and Patients-nonTI groups. **D** Cell correlation analysis (*p* < 0.05) of patients in tumor-infiltrating groups (Patients-TI). **E** Gene hazard ratios. Each gene signature for a digestive cancer (columns) is represented by hazard ratios in the treatment arms (rows) after dichotomization within the clinical trial. Vertical jitter distinguishes the overlapping dots. Hazard ratio < 1 (left of vertical lines) indicates greater PFS. The mean hazard ratio and one-sided *p* values are shown from a one-sample *z*-test of hazard ratios for genes in the signature, highlighted with associated data in red when Bonferroni-adjusted *p* < 0.05

We investigated the differential gene expression between Patients-TI and Patients-nonTI and identified genes with significant differential expression in each tumor type. Those genes made up the gene expression signature of tumor-infiltrating TCR groups for each tumor type. Three genes, CD8A, CRTAM, and EOMES, were consistently upregulated in all six tumor types (Fig. 5A). Using GO enrichment analysis (gene set enrichment analysis, GSEA), we found that the high expression genes were mainly enriched in immune activation-related pathways, such as GO0002253: activation of immune response, and GO0042110: T-cell activation (Fig. 5B). The proportion of CD8 T cells in the Patients-TI group was higher than that in the Patients-nonTI group (Fig. 5C). CD8 T cells were significantly positively correlated with T-cell regulatory (Tregs), follicular helper T cell, and M1 macrophages, and significantly negatively correlated with M0 macrophages, resting memory CD4 T cell, and activated mast cells (Fig. 5D). The proportion of macrophages M1, follicular helper T cells, and activated CD4 memory T cells in the Patients-TI group were higher than that in the Patients-nonTI group (Figure S5). Such evidences indicated that the patients with tumor-infiltrating TCR expansion were indeed in an immune-activated state.

These immune cells have strong antitumor activities and may be effective responders to immunotherapy. Using a published approach to analyze the correlation between the highly expressed genes and immunotherapy response (Wu et al. 2020), we found that patients with high expression genes (25 genes with *p* < 0.05 and the maximum logFC) in the gene signatures of tumor-infiltrating TCR groups tended to have longer PFS (Fig. 5E, S6), indicating that patients in these tumor-infiltrating TCR groups might benefit from immunotherapy.

### Viral specificity group inferences from HLA tetramer datasets

**Fig. 6:**
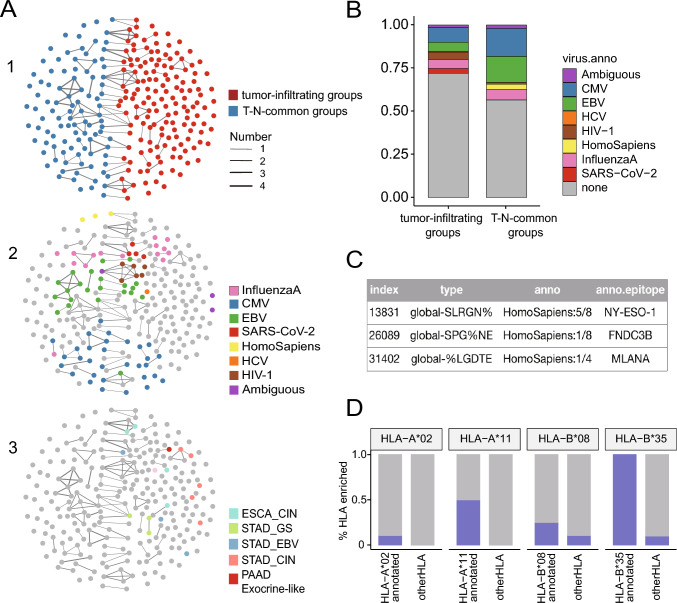
The virus annotation of those clone expansion groups. **A** Network analysis of 249 specificity groups annotated with tumor-infiltrating groups, CDR3β sequences that from HLA tetramers and tumor subtypes. Each dot represents a specificity group, and the edges indicate the presence of identical CDR3β sequence(s) shared across the two specificity groups. **B** Statistical plot of annotation to the tetramer data. EBV (green), CMV (blue), influenza (pink) and SARS-CoV-2 (red) antigens. **C** The annotated antigen from homo. **D** Percentage (%) of HLA-A*02, HLA-A*11 or HLA-B*08, HLA-B*35 tetramer-annotated specificity groups with significantly enriched the HLA-A*02, HLA-A*11 or HLA-B*08 and HLA-B*35 supertype alleles, respectively. Specificity groups annotated with tetramers of other HLA alleles (other tetramers) were included for comparisons

The publicly available HLA tetramer database (VDJdb) was used to annotate the potential antigens of the 249 clonal-expanded TCR groups defined by GLIPH2 (Fig. 6A). Among these 249 groups, 150 were tumor-infiltrating groups, whereas the rest were T-N-common groups. Both the tumor-infiltrating and T-N-common groups contained some groups that targeted virus antigens, the former having a lower proportion than the latter (Fig. 6A, B). Three groups targeted by human-derived antigens: NY-ESO-1, FNDC3B, and MLANA, were identified (Fig. 6C). We have performed differential mRNA expression analysis of NY-ESO-1, FNDC3B, and MLANA, which are the most common antigens among the tumor-specific groups. We found that NY-ESO-1 was barely expressed in our samples, while FNDC3B and MLANA were expressed at similar levels between the Patients-TI and Patients-nonTI groups. Therefore, we did not observe any significant difference in the expression of these antigens between the PS and PNS samples. Network analysis revealed that 44 of 48 (91.6%) groups in the 81 virus-annotated clone expansion groups were connected to the groups annotated with the same tetramer (Figure S7).

To validate the accuracy of HLA background annotation using tetramers, the enrichment of HLA alleles within the tumor-infiltrating TCR groups was examined. We focused on the HLA-A*02, HLA-A*11, HLA-B*08, and HLA-B*35 supertypes, as they were the most abundant in the dataset (Figure S7). We hypothesized that if a tumor-infiltrating TCR group was annotated by an HLA/peptide tetramer, the likelihood of observing enrichment of HLA alleles belonging to the same supertype defined by GLIPH2 should be higher. The results confirmed this hypothesis: 10%, 50%, 25%, and 100% of the tumor-infiltrating groups annotated with HLA-A*02, HLA-A*11, HLA-B*08, and HLA-B*35 tetramers, respectively, were enriched in their respective HLA contexts, which were higher than the percentage of any other tetramers (Fig. 6D). Therefore, the enrichment of a specific HLA allele within tumor-infiltrating groups is consistent with HLA-based restriction of binding between antigens and TCRs.

### Multidimensional integration of tumor-infiltrating TCR groups

Combined with the paired-chains TCR sequences reported in previous studies (Thorsson et al. 2018; Pai et al. 2023) and the paired-chains TCR data obtained from peripheral blood mononuclear cells (PBMCs) of pancreatic cancer patients in our laboratory, we obtained 184 complete CDR3α and CDR3β paired-chains TCR sequences (Table S5). We analyzed the conservation of CDR3β sequences in TCR groups with paired-chains sequences and enriched gene mutations and expression. It can be observed that the front and back parts of the CDR3β sequence are mostly conservative sequences, with the main changes concentrated in the middle section (Figure S8). We integrated all the results of mutation enrichment, subtype enrichment, gene markers, antigen annotation, and corresponding HLA in the group (Table S6). The TCR group in these results may be recognizing a neoantigen encoded by that mutation, presented on that HLA.

## Discussion

Our study comprehensively analyzed the TCR sequences of patients with digestive tract tumors, suggesting that the TCRs in the tumor microenvironment can form a group based on sequence similarity. There was heterogeneity between groups, reflecting the characteristics of different tumor mutation, gene marker and antigens. High expression of immune-activated genes in patients who had highly clonal-expanded and tumor-infiltrating TCRs was associated with a better prognosis in immunotherapy. The findings of these TCRs provided some information about the tumor microenvironment, reflected the immune response status of patients with digestive tract tumors, and have certain potential significance for clinical treatment and diagnosis.

These V and J genes that make up TCR tend to be highly expressed in both cancer and adjacent tissues (Jin et al. 2018). This indicates that these genes are common genes in tumor and normal tissues. Previous studies have shown that the HLA and CDR3 had a preference; a set of CDR3β sequences might have the same or similar antigen specificity, that is, they can recognize the same or similar peptide-MHC complexes (Chiou et al. 2021; Day et al. 2011; Madi et al. 2017; Madura et al. 2019). The preferential usage of these CDR3β sequences might be influenced by factors such as positive and negative selection during T-cell development, expansion and differentiation of T cells in peripheral tissues, and the function and survival of T cells in the tumor microenvironment. In addition, patients with high TCR diversity had worse prognosis for stage I STAD. This suggests that the decoupling of TCR binding and T-cell activation in tumors may be another mechanism of pathogenicity, as the natural process of specific responses to tumor-associated antigens (TAAs) is suppressed during tumor development. A previous study reported the opposite result, in which high TCR diversity was accompanied by favorable survival (Valpione et al. 2021). This discrepancy with previous results might be because different treatment methods for different stages of patients cause different immune responses in the body or because other factors affect TCR diversity, such as the tumor microenvironment, PD-1/PD-L1 signaling pathway, and T-cell memory subtypes.

The prognosis of patients in the Patients-TI and Patients-nonTI group only showed significant differences in STAD. We found that there was a significant deviation in the sample size between the Patients-TI and Patients-nonTI groups in five cancers except STAD, which may lead to inaccurate results. To remove this bias, we randomly selected a sample of patients in the Patients-nonTI group who had the same number as the Patients-TI group, and randomized 100 times to compare the significance of the difference in survival curves between the two groups. We found that in ESCA, LIHC, COAD, READ, and PAAD, there were 5 out of 100 cases with *p* < = 0.05, while in STAD, there were 46 cases with *p* < = 0.05. This result indicates that there is no significant difference in survival among cancers other than STAD. Consistent with previous results. We analyzed the reasons for the differences between the two groups of STAD from a molecular mechanism perspective. We performed differential gene expression and enrichment analysis on patients in the Patients-TI and Patients-nonTI groups, and the results showed that the Patients-TI group was enriched in cell activation involved in negative regulation of toll-like receptor signaling pathway, negative regulation of inflammatory response to antigenic stimulus, negative regulation of type I interferon production. These pathways were not enriched in other tumors in the Patients-TI group.

Tumor-infiltrating groups are enriched for different gene mutations, and different mutation-enriched TCR groups may correspond to neoantigen generated by the corresponding mutations. We can obtain antigenic epitopes that respond effectively from TCR analysis, and inferred the tumor dominant antigens that activate T cells and generate cascade responses. The enrichment of gene mutations and tumor subtypes we found here may be due to differences in the immune microenvironment as a result of neoantigens caused by mutations, or to differences in mesenchymal stromal cells between tumor subtypes, which may lead to differences in the T-cell population.

In a previous study on sharing TCRs in non-small-cell lung cancer, TMEM161A was identified as a novel tumor-associated antigen; its overexpression in tumors and its cross-reactive epitopes were from EBV LMP2A and *E. coli* (Chiou et al. 2021). We found instances in which TCRs exhibited cross-reactivity to both tumor and microbial antigens. Therefore, this seems to be a possible explanation for reports of pathogen-specific T-cell infiltration into tumors (Scheper et al. 2019; Rosato et al. 2019; Simoni et al. 2018). Our research showed T-cell specificities for TAAs and pathogen-derived antigens were not mutually exclusive. Maintaining a broad T-cell repertoire to defend against pathogens may largely depend on the cross-reactivity of TCRs (Su et al. 2013). In this study, three tumor antigens were annotated. NY-ESO-1 is a widely studied novel antigen that could become a target for the treatment of various cancers (Thomas et al. 2018; Gyurdieva et al. 2022). The proportion of cross-reactivity between tumor antigens and viral antigens in the tumor-infiltrating group was lower than that in the T-N-common groups. A previous report has shown that EBV was rarely detected in lung cancer (Kheir et al. 2019). Pathogens can serve as the basis for immunotherapy, and the gut microbiome is a key determinant of the cancer immunotherapy response (Routy et al. 2018; Matson et al. 2018; Gopalakrishnan et al. 2018). In pancreatic cancer, it has been observed the patients with the longest survival after surgery have a unique microbiome composition (Riquelme et al. 2019).

We identified genes that are involved in HLA antigen presentation, such as HLA class I, Beta 2, Macroglobulin, TAP1, TAP2, ERAP1, PSMB8, PSMB9 and PSMB10 (Surmann et al. 2015; Seliger et al. 2017, 2000; Atkins et al. 2004; Scupoli et al. 1996; Li et al. 2016). The downregulation of HLA genes or other genes involved in antigen presentation could affect the TCR repertoire and the response to immunotherapy. We have performed differential expression analysis on the Patients-TI and Patients-nonTI groups. We did not find any significant downregulation of these genes between the two groups. Therefore, we did not observe any evidence of HLA antigen presentation impairment in our samples. Therefore, we do not think that this factor explains the difference in the number of PS and PNS patients. Regarding the HLA genes that were found in our dataset but not in VDJdb, we acknowledge that this could limit the accuracy of our antigen annotation for the TCR group, but we believe that this is a minor issue, as most of the HLA genes in our dataset are well represented in VDJdb. Moreover, the PS and PNS patients were defined by the TCR group rather than the antigen annotation.

Our study has some limitations. Using RNA-Seq data to identify CDR3 sequences may not capture the full diversity of the TCR repertoire, as some clones may be missed due to low expression or sequencing depth. However, we believe that our approach still provides a valuable overview of the TCR landscape in tumor tissues, as it does not require additional PCR amplification or sequencing of the TCR template. The GLIPH2 algorithm only inferred T-cell specificity based on TCR β sequences, i.e., it captures only a fraction of the input sequences. Although our study only analyzed the immune repertoire of digestive tract tumors, many of the shared TCRs found in this study were not limited to digestive tract tumors. We obtained a set of tumor-infiltrating shared TCRs, but they were not functionally validated; and these TCRs does not have necessarily an antitumor effect (if regulatory T cells). Therefore, our findings need further validation on a more multi-dimensional dataset, and the potential of these TCR sequences as immunotherapy targets or biomarkers should be assessed in animal models and clinical trials. T-cell receptor engineered T cells (TCR-T) therapy is a novel adoptive cell therapy method that mainly uses gene editing technology to introduce TCR genes that can specifically recognize tumor antigens into patient T cells, allowing them to express exogenous TCR and thus have specific activity in killing tumor cells. With these TCR sequences, we can optimize the codons and find DNA plasmids that can express the same peptide sequence, then transfect them into T cells for expression. Infuse T cells back into mice for tumor cell-killing experiments. At present, our laboratory is also conducting relevant experiments.

In conclusion, our study provides new insights and pipeline to understand the immune status and response of patients with digestive tract tumors and lays the foundation for the development of personalized and precise immunotherapies.

### Supplementary Information

Below is the link to the electronic supplementary material.

Supplementary file1 Figure S1. (A) The pipeline of identify groups. (B) Distribution of the most frequent TRBJ gene in all clone expansion groups. The y axis shows the number of groups. The x axis shows the TRBJ genes shared by those groups. (C) The class of HLA in tumor-infiltrating groups. (D) The HLA statistics of all patient. (EPS 1898 KB)

Supplementary file2 Figure S2. The result of Cox proportional risk model applied to all six digestive tract cancer patients. (EPS 3032 KB)

Supplementary file3 Figure S3. The survival plot of all cancer patients between the groups of Patients-TI and Patients-nonTI. (EPS 2635 KB)

Supplementary file4 Figure S4. The type of cancers in Patients-TI and Patients-nonTI groups. The x axis shows six cancer and y axis shows the number of patients in each group. (EPS 353 KB)

Supplementary file5 Figure S5. The boxplot of immune cells proportion. (EPS 1045 KB)

Supplementary file6 Figure S6. The survival plot of two groups in relevant arms based on high expression genes in six cancer types. (EPS 1539 KB)

Supplementary file7 Figure S7. The virus annotation in clonal-expansion groups. (EPS 2068 KB)

Supplementary file8 Figure S8. The sequence logos plot in identical gliph2 clusters. (EPS 1399 KB)

Supplementary file9 Figure S9. The heatmap of PAAD subtypes. (EPS 2798 KB)

Supplementary file10 Table S1. All BAM files of digestive tract tumors downloaded from the TCGA. (XLSX 228 KB)

Supplementary file11 Table S2. The statistics of all digestive tract cancer. (XLSX 10 KB)

Supplementary file12 Table S3. The statistics of tumor-infiltrating TCR groups. (XLSX 25 KB)

Supplementary file13 Table S4. The statistics of enriched HLA in each group. (XLSX 15 KB)

Supplementary file14 Table S5. The paired chain TCR of integrated. (XLSX 15 KB)

Supplementary file15 Table S6. The integration of mutation enrichment, subtype enrichment, gene markers, antigen annotation, and corresponding HLA in the group. (XLSX 19 KB)

## Data Availability

All data, codes, and materials used in the analyses are available from the corresponding author upon reasonable request.
